# Use of a sequential multiple assignment randomized trial to test contingency management and an integrated behavioral economic and mindfulness intervention for buprenorphine-naloxone medication adherence for opioid use disorder

**DOI:** 10.1186/s13063-023-07102-9

**Published:** 2023-03-29

**Authors:** Samuel C. Peter, James G. Murphy, Katie Witkiewitz, Sarah B. Hand, Fridtjof Thomas, Karen Chandler Johnson, Ronald Cowan, Matt Harris, Karen J. Derefinko

**Affiliations:** 1 Department of Psychology, Durham VA Medical Center, 508 Fulton Street, Durham, NC 27705 USA; 2 Department of Psychology, The University of Memphis, 400 Innovation Drive, Memphis, TN 38152-6400 USA; 3 Department of Psychology, University of New Mexico, Logan Hall, Albuquerque, NM 87131-0001 USA; 4 Department of Preventive Medicine, University of Tennessee Health Science Center, 66 N Pauline St, Memphis, TN 38163 USA; 5 Department of Psychiatry, University of Tennessee Health Science Center, 920 Madison Avenue, Memphis, TN 38163 USA; 6 Boyd Center for Business and Economic Research, University of Tennessee, 1000 Volunteer Boulevard, Knoxville, TN 37996 USA

**Keywords:** Behavioral economics, Contingency management, Substance-free activities, Brief motivational intervention, Opioid use disorder, Sequential Multiple Assignment Randomized Trial

## Abstract

**Background:**

Buprenorphine-naloxone is a medication shown to improve outcomes for individuals seeking treatment for opioid use disorder (OUD); however, outcomes are limited by low medication adherence rates. This is especially true during the early stages of treatment.

**Methods:**

The present study proposes to utilize a sequential multiple assignment randomized trial design to compare two psychological interventions targeting buprenorphine-naloxone adherence: (1) contingency management (CM) and (2) brief motivational interviewing plus substance-free activities session plus mindfulness (BSM). Participants will be *N* = 280 adults who present to a university-based addictions clinic seeking treatment for OUD. Participants will be randomized to condition to receive 4 sessions of their assigned intervention (CM or BSM). Participants who are adherent, defined as attending physician appointments and having buprenorphine present in urine toxicology, will enter maintenance intervention for an additional 6 months. Those who are not adherent will be re-randomized to receive either the other intervention or both interventions. Follow-up will occur at 8 months post-randomization.

**Conclusions:**

This novel design will examine the benefit of sequential treatment decisions following non-adherence. The primary outcome of this study is buprenorphine-naloxone medication adherence, as assessed by physician visit attendance and presence of buprenorphine in urine. Results will elicit the relative efficacy of CM and BSM compared to one another and whether keeping the initial treatment approach when adding the alternative approach for initially non-adherent individuals is beneficial.

**Trial registration:**

ClinicalTrials.gov NCT04080180

**Supplementary Information:**

The online version contains supplementary material available at 10.1186/s13063-023-07102-9.

## Background

Buprenorphine-naloxone, an opioid agonist–antagonist, is frequently used as a medication to treat opioid use disorder (MOUD). Recent reviews show that buprenorphine-naloxone is an effective treatment for opioid detoxification, harm reduction, and maintenance therapy in inpatient and outpatient settings [1, 2]. Compared with methadone, buprenorphine-naloxone demonstrates higher abstinence rates, less patient burden, better health-related quality of life, and improved relationships [3, 4].

Unfortunately, MOUD adherence is very low [5, 6]. A typical buprenorphine-naloxone treatment regime includes initial weekly visits to the physician for approximately 1 month to establish dosage and adherence to buprenorphine-naloxone protocol, followed by monthly visits thereafter for maintenance therapy [7]. Our research in a rural Tennessee outpatient setting (*N*= 87) indicated that over half of individuals who began buprenorphine-naloxone engaged in illicit opioid use within the first two office visits, and the majority of these individuals did not return to buprenorphine-naloxone treatment [8]. Several other research studies have also documented these low MOUD adherence rates [5, 9, 10] particularly early in treatment [11].

Remarkably little research has been conducted to address adherence in MOUD patients. The American Society of Addiction Medicine’s *National Practice Guideline for the Use of Medications in the Treatment of Addiction Involving Opioid Use *[12] recommends that psychosocial intervention be included to assist individuals in coping with the transition from illicit opioids to MOUD. As cited in the report, Contingency Management (CM) and other cognitive and behavioral therapies (CBT) have been widely studied across different forms of substance use disorders, but effectiveness of these forms of treatment specifically for buprenorphine-naloxone adherence is less well understood [13].

### Contingency management

Based on operant conditioning, CM is a behavioral treatment where prizes or vouchers are provided to patients based upon proof of a desired behavior such as abstinence or adherence to a medication. Importantly, CM may be ideal for individuals with low motivation to change or limited environmental support for abstinence or treatment engagement given that it provides an immediate tangible reward such as money or a prize that is contingent upon achieving the target outcome (e.g., chemically verified abstinence or medication compliance) [14, 15]. A meta-analysis of 30 studies that evaluated the use of CM to promote opioid abstinence in patients receiving methadone showed that CM resulted in a greater number of drug-negative urinalysis screens (effect size *r*= 0.25) compared to a control group and that this effect was stronger for immediate rewards [14]. Other meta-analyses have shown that CM is not as effective at improving clinic attendance (*r* = 0.15) [16] but has greater efficacy for opioid-specific abstinence (*d* = 0.65) than general substance use abstinence (*d*= 0.42) [17]. Interestingly, larger effect sizes appear to be associated with *shorter*treatment duration, suggesting that the effects of CM may diminish over time [17].Although no studies have explored the use of CM for buprenorphine-naloxone adherence, across other forms of MOUD (e.g., methadone), the effect size of CM on adherence was found to be medium to large (*d*= 0.75) and it is thus a promising approach [18].

#### Cognitive behavioral therapy

Research on CBT for substance use disorder has predominantly examined effects of CBT on substance use abstinence, not MOUD adherence. CBT provides individuals with strategies designed to help the patient identify high-risk situations and triggers and to promote the use of effective coping strategies to avoid substance use in high-risk situations [19–21]. In a review of adjunctive CBT intervention for those in buprenorphine treatment [22], only 11 RCTs were identified, and evidence from the low risk of bias studies therein indicated that adding psychosocial interventions to buprenorphine treatment *does not* significantly improve substance use outcomes. However, these adjunctive treatments varied widely in method and study power, with outcomes that were self-reported or did not actually assess medication compliance, a known predictor of successful treatment outcomes.

Substance use disorder treatment compliance may also be enhanced by other interventions that include a reinforcement focus [23]. Behavioral economic theory suggests that individuals generally make decisions in a manner that maximizes overall reward or utility, but that this “rationality” is heavily constrained by a characteristic bias towards immediate reward, and a tendency to discount the value of delayed rewards. There are reliable individual differences in the degree to which delayed rewards are discounted, and steep delayed reward discounting can contribute to a consistent preference for the immediate reward associated with drug use relative to the delayed reward associated with many substance-free activities [24]. Likewise, deciding to abstain from drugs or to engage with treatment requires an ability to forgo the immediate reward associated with drug use and to organize patterns of behavior around the pursuit of future social and health benefits associated with treatment and reductions in drug use [25–29]. Another key tenet of behavior economic theory is that levels of drug use are generally inversely related to levels of environmental reward and that interventions that increase access to substance-free reward will reduce substance use [23].

Murphy and colleagues developed a brief intervention called the substance-free activity session (SFAS) which uses a motivational interviewing approach to target the behavioral economic mechanisms of substance-free reinforcement and delay discounting [30]. Specific session elements include a discussion of future goals, the benefits of achieving those goals, and the congruence between substance use and achieving those goals. The SFAS includes personalized feedback on recent time allocation across a variety of activities (e.g., drinking/drug use, exercising, time with family, volunteering) to highlight potential discrepancies in how the individual spends their time and personal goals or values [31, 32]. This intervention was associated with significant reductions in drinking and related problems in two different trials with non-treatment-seeking college students [30, 31].

In addition to the focus on goals and substance-free activities, strategies that address MOUD adherence should consider the issues specific to opioid withdrawal and initiation of buprenorphine-naloxone. MOUD initiation is fraught with opioid craving and the unpleasantness of withdrawal from the illicit opioid, an unavoidable outcome. Therefore, individuals in MOUD may benefit from an approach that helps them deal with those unpleasant experiences. Witkiewitz et al. describe how mindfulness techniques can be practiced with the patient to develop their ability to observe their emotional and physiologic state without reactivity, thereby providing space to engage in behavioral responses that are more aligned with patient values and to engage in coping with craving and urges to reduce substance use [33]. The “Stop Observe Breathe Expand Respond (SOBER) Breathing Space” exercise is a brief mindfulness practice that is widely accepted by patients, easy to remember, and accessible in a variety of situations and contexts, [34] making it a beneficial component to reduce risk of illicit opioid use in high-risk situations.

### Comparing CM and CBT

Although both CM and CBT methods have the potential to improve medication adherence, CBT-based strategies have greater potential for dissemination than CM. Although CM is widely accepted as an efficacious treatment for substance use disorder and remains an accepted program for substance use cessation in the Veteran’s Administration system [35], this strategy has not often gained a foothold in community settings [36].

In addition, it is possible that these complimentary strategies are differentially effective at different treatment stages. Evidence suggests that CM reward effects diminish, whereas CBT strategies gain momentum over time [37, 38]. One study of individuals with cocaine dependence (*N*= 120) directly examined the long-term abstinence outcomes of CM vs. CBT over a period of 12 months [37]. Cocaine urinalysis results indicated that for those in the CM group, abstinence *declined* substantially over time (63%, 40%, and 44%, across 17, 26, and 52 weeks, respectively). Conversely, the CBT group showed significant *increases*in abstinence over the follow-up time periods (33%, 64%, and 81%, respectively) [37]. Another study comparing these same treatment conditions was conducted with individuals with concomitant cocaine use who were receiving methadone maintenance [39]. Although CM was found to be the superior treatment at 16 weeks, the percentage of CBT group participants with cocaine-free urine samples (53%) significantly exceeded percentages in CM (47%) at 26 weeks [39].

While these long-term abstinence results are promising, the traditional CBT for substance use tested in these studies did not explicitly address mindfulness, delay discounting, or engagement with substance-free alternative activities and did not address medication adherence. Based on behavioral economic theory, brief motivational interviewing plus substance-free activities session plus mindfulness (BSM) could alter time orientation to value the future more and changing behavior to pursue drug-free rewards and manage cravings that are prevalent during treatment initiation. BSM is likely to be a critical mechanism to support medication adherence and promote recovery and can be delivered efficiently in a brief format.

At this point, there is considerable evidence of the effectiveness of CM and the CBT strategies in BSM for substance use abstinence, but less compelling or complete evidence of how these methods address adherence to buprenorphine-naloxone, the current state of the science treatment for OUD [1, 2]. Given that adherence to buprenorphine-naloxone is closely related to long-term treatment success [40], the specific and objective evaluation of buprenorphine-naloxone adherence is paramount to evaluating intervention utility.

Our research team seeks to compare the effectiveness of these two different interventions for MOUD adherence by conducting a sequential multiple assignment randomized trial (SMART) to assess sequential, individual, and combined effects of these two forms of treatment across MOUD initiation and maintenance. We will randomize 280 buprenorphine-naloxone initiating patients to receive either CM or BSM for their first 4 physician visits for MOUD. Adherence will be established via attendance and toxicology screen. Participants who are adherent will enter maintenance intervention (remaining in their assigned intervention condition for up to 6 months). Those who are not adherent for the initial intervention period will be re-randomized to receive either the other intervention or a combined intervention and will then continue to receive this intervention for an additional 6 months.

#### ***Hypotheses***

Hypothesis 1: Due to its potency with immediate and tangible rewards [35], we predict that a higher percentage of those in the CM condition will be initially adherent than those in BSM. Hypothesis 2: Based upon evidence of diminished effects of CM over time [17, 38], we predict that those who remain in CM (vs. BSM) will show decreases in adherence during the 8-month follow-up. Hypothesis 3: We predict that the combined CM + BSM will have the largest treatment effect among those re-randomized given the greater intensity of intervention and presence of internal and external motivators [13].

## Methods

This study has been registered with ClinicalTrials.gov (NCT04080180), has Institutional Research Board approval, and is monitored by a Data and Safety Monitoring Board. Informed consent will be obtained from all participants before any research activities occur.

### Trial design

**Fig. 1:**
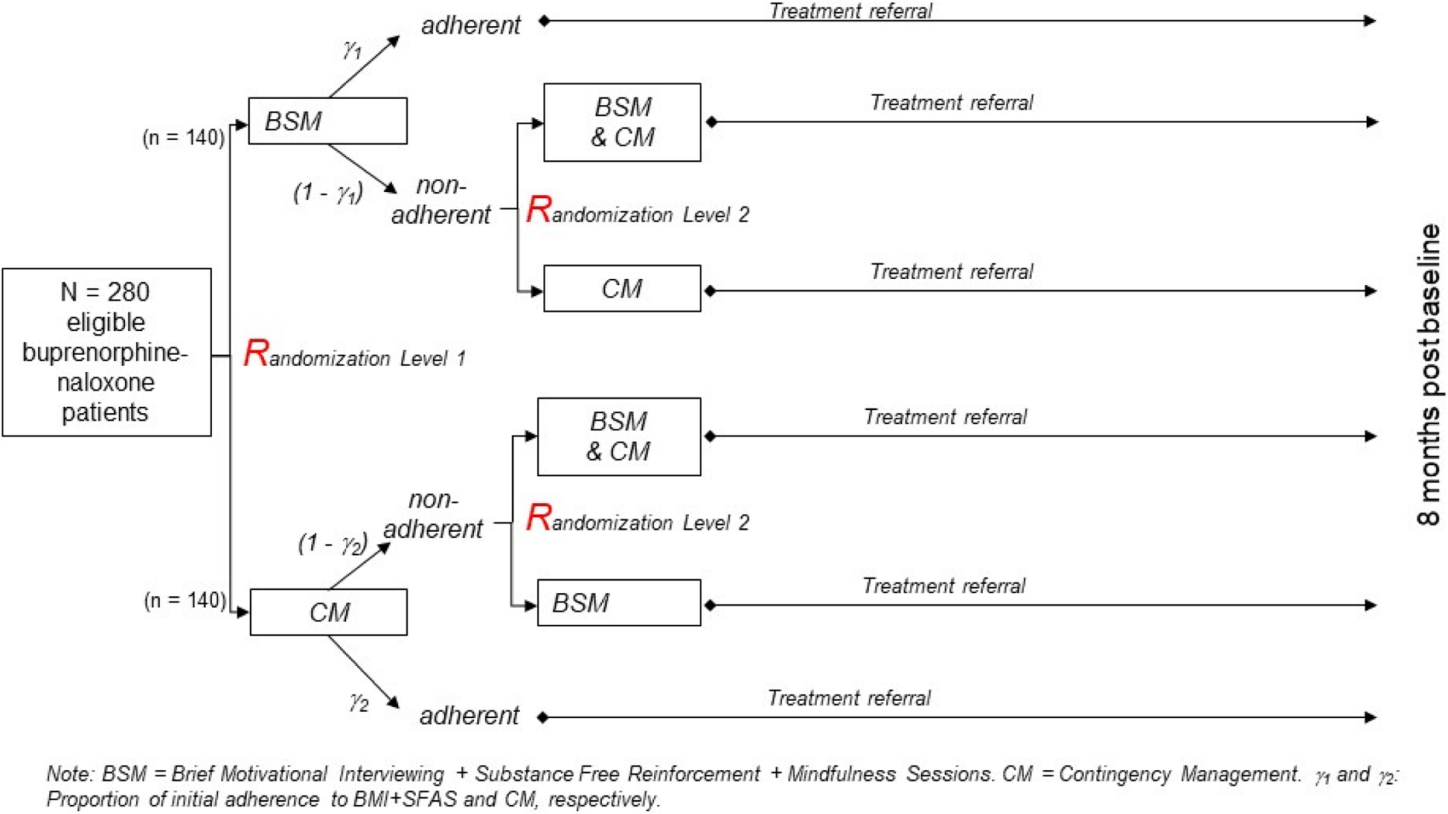
Sequential Multiple Assignment Randomized Trial (SMART) design

This is randomized superiority clinical trial design with individual parallel block-randomization (1:1) to 2 active intervention groups at the first stage (Stage 1), CM or BSM, and subsequent (Stage 2) randomization for initially non-adherent participants (known as SMART design). Figure 1 presents the participant flowchart, including information on the timing of re-randomization, length of intervention, and follow-up periods.

### Participants

This study is being conducted within an established university-based addictions clinic in the southeast USA. Participants (*N* = 280) will be patients from the community seeking treatment at this clinic. To be eligible for participation, participants must present with symptoms of OUD and be eligible for receipt of buprenorphine-naloxone medication (e.g., Suboxone, Bunavail, Zubsolv), as determined by the director of the medical center and co-investigator of this study. Participants must also be at least 18 years old, have the ability to understand consent procedures, and have access to a telephone. Participants are allowed to be in concomitant care during study participation.

Due to the high likelihood of relapse during MOUD treatment initiation, we are recruiting patients at their first visit to the clinic and providing adjunctive intervention (CM or BSM) only for the first 4 visits for MOUD with the addiction medicine physician. However, because there is risk of illicit opioid use for individuals who switch MOUD providers, we consider all new patients to the clinic eligible, regardless of prior experience with MOUD. Those who are non-adherent will be re-randomized (described below).

### Recruitment, screening, randomization, and procedure

We will engage with referred MOUD patients at the time of their initial visit to the university-based addictions clinic. Interested patients will be informed about the study in a private room and, if eligible, will be consented and randomized to condition.

Randomization for Stage 1 and Stage 2 is a 2-arm, parallel, random assignment with 1:1 allocation ratio using block-randomization (blocks of 4) developed by the study statistician with separate blocks for males and females to avoid coincidental assignment imbalance over time and to ensure that males and females are comparatively represented in both of the respective randomization points. Randomization sequence will be stored in a database where the sequence is concealed until participants are enrolled and interventions are assigned by the study coordinators. Buprenorphine providers and the outcomes assessor will be blinded to condition.

Following randomization, participants will start their assigned intervention at their physician visits for MOUD. Notably, patients’ MOUD visit schedules vary during treatment initiation and, depending upon circumstances, can occur weekly, bi-weekly, or monthly.

At each of these 4 MOUD physician visits (known as treatment initiation regardless of time period between visits), participants will complete assessments, then engage in intervention activities. Those who are adherent to buprenorphine-naloxone (i.e., attend physician visits and demonstrate presence of buprenorphine in urine screen) during this 4-visit period will remain in their intervention condition for up to 6 months (maintenance intervention). A final assessment will be conducted at 8 months from Stage 1 randomization.

Those who are not adherent to buprenorphine-naloxone, defined as missing their physician visit or absence of buprenorphine in urine screen, will be re-randomized to either switch to the other intervention condition or add the other intervention (CM + BSM combined). We expect that about 50% of the participants will be initially non-adherent and will qualify to be re-randomized. For re-randomized participants, intervention will start from the beginning.

### Retention plan for enrolled participants

To encourage participation in the follow-up assessments, all participants will receive $50 gift certificates to Amazon for the completion of each of the assessment contacts. All participants will receive appointment reminders via their preferred modality (text message, phone call, or email) the day before their appointments. For participants who miss an appointment or are lost to follow-up, we will attempt to contact them at least three times a week when they are in an open visit window (i.e., due for a visit). We will use multiple different modalities including phone call, text messages, email, and contacting the participant’s alternate contact person. We will also attempt to contact the participant at different times of the day.

### Intervention conditions

#### CM

Those randomized to the CM intervention will be allowed to spin a virtual wheel upon a positive buprenorphine screen. The wheel spin will allow a 60% chance of receiving a $50 gift card, a 25% chance of a $75 card, and a 25% chance of a $100 card. This contemporary technique has been validated in the CM literature and is believed to enhance reward salience through the intermittent receipt of high reward value [41].

### BSM (Table 1)

**Table 1:** Components of typical BSM session

Component/topic	Description/example therapist prompts
Short- and long-term goals	
Identifying goals	What are your goals for this month…for the next 5 years? What would you like to have accomplished?
Requirements to achieve goals	What would you need to do to achieve those goals?
Relation between OUD and goals	Examine the potential role of illicit opioid use in jeopardizing these goals
Reward bundling	Aggregate global day-to-day choices and activities into cumulative, cohesive patterns that relate to personally relevant long-term health or social outcomes. A behavior with immediate but low reward value (i.e., taking a daily dose of buprenorphine-naloxone) in the short term may have higher reward value when it is framed as part of a pattern of achieving a valued long-term outcome (i.e., steady employment at a desired job)
Episodic future thinking	An experiential intervention that prompts individuals to describe personal, emotional, and situational details of a valued future outcome in great detail (e.g., what it would be like to regain family trust or get a career-job)
Substance-free activities	Participants will be offered a menu of substance-free activities that has been developed in pilot work. Activities will be discussed, and participants will be asked to engage in selected activities as homework. Engagement in substance-free activities will be assessed at each subsequent visit
SOBER	Stop and see what happens Observe physical sensations and emotion regulation changes in the body Breathe by deliberately bringing attention to the breath Describe to the participant how to breathe in through their nose and out through their mouth slowly Expand awareness of the situation Respond mindfully (versus reacting)

*Note.* Each BSM session is completed in approximately 45 min

Those randomized to the BSM will engage in a weekly discussion centered around increasing engagement in substance-free activities that are enjoyable and/or consistent with the individual’s goals or values. A related focus is enhancing the salience of future goals and of the association between daily patterns of behavior and achieving these outcomes, as well as the potential impact of relapse on those goals. This includes an episodic future thinking exercise where the individual vividly imagines and describes a positive future event associated with treatment adherence and achieving sobriety, as well as ongoing planning to engage in specific substance-free activities, and the SOBER breathing space exercise [34, 42, 43]. Each session of this 4-session intervention will take 45 min. Further descriptions of these intervention components can be found in Table 1.

## Assessment components

Assessments will be administered by study coordinators and research assistants. All research staff will be trained in general clinical interviewing along with specific training for each measure that will be administered. A staff member trained in data quality assurance will check entered data weekly for trends in missing data or inconsistencies. The database will also be programmed to show an error message if a data point is missing during live data entry.

### Demographics

Demographics will include age, contact information, gender, race, ethnicity, dependency status, marital status, education, and household income. These characteristics will only be assessed at baseline.

### Contact information

Contact information will include name, address, email address, and emergency contact persons and contacts for retention purposes. Participants will provide updated contact information at each study visit to aid in retention.

### Opioid use disorder

Opioid use disorder will be assessed at baseline using symptom criteria from the Diagnostic and Statistical Manual of Mental Disorders-5^th^edition [44].

### Delay discounting

Delay discounting will be measured using the Money Choice Task [45], a 27-item questionnaire which asks the participant to decide between immediate, small rewards, and delayed, but larger rewards. The individual’s “discount rate” determines the steepness of the reduction in present value of delayed rewards. As the discount rate increases, the duration of the window of vulnerability increases, as does the strength of preference for the impulsive choice within the window. A participant’s discounting curve may be calculated according to the following function: *V* = *A*/(1 + *kD*) where *V* is the present value of the delayed reward *A* at delay *D*, and *k* is the rate of discounting. *k* typically falls between 0.0 and 0.5, with smaller values indicating a lack of discounting and preference for delayed rewards and higher values indicating strong discounting and a preference for immediate rewards. Thus, higher values of *k*are indicative of high levels of impulsivity. The Money Choice Questionnaire has demonstrated good test–retest reliability over 3 years and external validity as evidenced by relations to impulsive behaviors, such as crime and substance use [45, 46].

### Opioid Purchase Task [47]

The Behavioral Economic framework suggests that addiction is based on a recurrent and persistent pattern of elevated motivation for drugs [24]. Reinforcing efficacy of the substance is often quantified using hypothetical purchase tasks in which individuals provide values for the drug of choice across a range of escalating prices (i.e., $0, $0.25, $0.50, $1, $1.50, $2, $2.50, $0.3, $0.3.50, $4, $4.50, $5, $6, $7, $8, $9, $10, $11, $12, $13, $14, $15, $20, $25, and $30). From these tasks, *demand and expenditure curves* can be plotted, which yield several indices of relative value including intensity of demand (consumption at lowest price, usually zero); breakpoint (price at which consumption first reaches zero); *O*_max_ (maximum expenditure); *P*_max_(price corresponding to maximum expenditure); and elasticity (i.e., relative change in consumption in response to change in commodity price) [48, 49]. The measurement of reinforcing efficacy using hypothetical purchase tasks among young adults has been validated using the Alcohol Purchase Task (APT) [50] to assess alcohol demand. Indices derived in this hypothetical task demonstrate good test–retest reliability [51] and are associated with demand indices derived from actual consumption choices in a laboratory setting [52]. They also evidence associations with alcohol problems beyond consumption [51] and predict treatment outcomes following brief drinking interventions [53, 54]. Modified hypothetical purchase tasks have been used to assess young adult marijuana demand [55], cigarette demand [56], and non-prescribed opioid demand [47]. The Opioid Purchase Task will be assessed at each visit.

### Engagement in substance-free activities [57]

This is a 47-item measure which asks participants to rate the extent to which the following factors have been helpful in their recovery from opioids either currently or during periods in the past when they stopped using. Items are scored on a 3-point Likert scale ranging from 1 = Not at all helpful to 3 = Very helpful.

### Brief Pain Inventory Short Form

The BPI Short Form [58] assesses pain severity and impact on daily life function. The short form is a 9-item measure that assessed multiple areas in a Likert scale, open-ended, and visual analog scale type questions. Research with chronic pain patients indicates that the BPI Short Form is internally reliable, test–retest reliable and is valid in terms of construct, convergent, and predictive validity [58].

### EQ5D5L

The 5-level EQ-5D version (EQ-5D-5L) [59] assesses quality of life across 5 dimensions: mobility, usual activities, self-care, pain/discomfort, and anxiety/depression. Each dimension is scored on a 5-point scale: 1 = no problems, 2 = slight problems, 3 = moderate problems, 4 = severe problems, and 5 = extreme problems. The patient is asked to indicate his/her health state by ticking the box next to the most appropriate statement in each of the five dimensions. This decision results in a 1-digit number that expresses the level selected for that dimension.

### Generalized anxiety disorder 2-item

The Generalized Anxiety Disorder 2-item (GAD-2) is a brief screening tool for generalized anxiety disorder [60]. The two items are scored on a 4-point scale ranging from 0 (Not at all) to 3 (nearly every day). The total score is obtained by adding the scores from the two items. Research suggests that the GAD-2 has a sensitivity of 86% and specificity of 83% for predicting generalized anxiety disorder.

### PEG for chronic pain

The Pain, Enjoyment, General Activity scale (PEG for Chronic Pain) [61] is a 3-item measure that assesses pain intensity and functional interference. It has demonstrated good reliability and validity in large samples of chronic pain patients in primary care, VA hospitals, and pain management centers [61]. The PEG is scored by averaging the items; scores range from 0 to 10. Its excellent psychometric properties and responsiveness to treatment compare favorably to other established measures such as the PROMIS and SF-36 pain scales [62].

### The Patient Health Questionnaire-2

The Patient Health Questionnaire-2 (PHQ-2) is a 2-item questionnaire that measures [63] the frequency of depressed mood and anhedonia over the past 2 weeks. The two items are scored on a 4-point scale ranging from 0 (Not at all) to 3 (nearly every day). The total score is obtained by adding the scores from the two items. Research suggests that the PHQ-2 has a sensitivity of 97.6% and specificity of 59.2% for predicting major depressive disorder.

### Tobacco, Alcohol, Prescription medication, and other Substance use

The Tobacco, Alcohol, Prescription medication, and other Substance use (TAPS) Tool [64] for substance use screening is a 4-item screen for tobacco, alcohol, illicit drugs, and non-medical use of prescription drugs, followed by a substance-specific assessment of risk level for individuals who screen positive. Items are scored on a 6-point Likert scale, ranging from 0 = never to 5 = daily. In a sample of 2000 medical patients, the TAPS Tool had a sensitivity of 0.93 (95% CI 0.90–0.95) and specificity of 0.87 (95% CI 0.85–0.89) for tobacco, and a sensitivity of 0.74 (95% CI 0.70–0.78) and specificity of 0.79 (95% CI 0.76–0.81) for alcohol [64].

### Timeline Followback

The Timeline Followback (TLFB) [65] is a method that can be used as a clinical and research tool to obtain a variety of quantitative estimates of marijuana, cigarette, and other drug use. The TLFB can be administered by an interviewer, self-administered, or administered by computer. It involves asking clients to retrospectively estimate their drug, marijuana, or cigarette use 7 days to 2 years prior to the interview date [66].

### Opioid Craving Symptoms [67]

Craving is a cardinal feature of substance use disorder and has been shown to predict future drug use. Because of this, craving is often assessed in treatment settings as a marker of risk for subsequent drug use. The *Opioid Craving Scale*, a modification of the Cocaine Craving Scale [68] consists of three items rated on a visual analog scale from 0 to 10: (1) How much do you currently crave opiates? (rated from *not at all* to *extremely*), (2) In the past week, please rate how strong your desire to use opiates has been when something in the environment has reminded you of opiates (rated from *no desire* to *extremely strong*), and (3) Please imagine yourself in the environment in which you previously used opiates. If you were in this environment today and if it were the time of day that you typically used opiates, what is the likelihood that you would use opiates today? (rated from *not at all* to *I’m sure I would use opiates*). This scale has demonstrated strong internal reliability, and greater craving was found to be associated with higher odds of prescription opioid use in the following week. For each one-unit increase on this scale, the odds of using opioids in the subsequent week was 17% higher [67].

### Perceived social support

Perceived social support [69] is assessed via a 40-item scale which asks about support from friends and family. Each item is scored as Yes/No/I Don’t Know. Three studies affirmed the internal reliability of the PSS and confirmed the validity of the measure via relations with measures of supportive relationships, positive life events, anxiety, and depression.

### Toxicology screen

The participant’s toxicology screen will be abstracted from the medical record.

### Attendance at clinic visit

Attendance at clinic visit is reported as yes or no for each visit window.

### Dosage of buprenorphine-naloxone prescribed

Dosage of buprenorphine-naloxone prescribed will be obtained from medical record at each visit.

### Buprenorphine-naloxone dose log (recorded by participant)

Self-reported adherence to buprenorphine will assessed by the participant reporting how many days they skipped their medication since their last buprenorphine care provider visit in conjunction with a calculation of the total number of days since their last buprenorphine care provider visit.

The full schedule of assessments and intervention delivery can be found in Table 2.

**Table 2:** SPIRIT figure—schedule of enrollment, interventions, and assessments

	Study period										
	Enrollment	Allocation	Post-allocation								Close-out
			Stage 1				Stage 2				
TIMEPOINT	− *t*_1_	0	*t*_1_	*t*_2_	*t*_3_	*t*_4_	*t*_5_	*t*_6_	*t*_7_	*t*_8_	*t*_9_
ENROLLMENT:											
Eligibility screen	X										
Informed Consent	X										
Allocation		X									
INTERVENTIONS:											
Stage 1 CM			X	X	X	X					
Stage 1 BSM			X	X	X	X					
Stage 2 Switch							X	X	X	X	
Stage 2 Combine							X	X	X	X	
ASSESSMENTS:											
Baseline											
Inclusion/exclusion	X										
Contact information	X		X	X	X	X	X	X	X	X	
Demographics	X										
Opioid use disorder	X										
Outcomes											
Toxicology screen			X	X	X	X	X	X	X	X	X
Clinic visit attended			X	X	X	X	X	X	X	X	
Moderators											
Opioid Cravings Scale	X		X	X	X	X	X	X	X	X	X
TAPS Tool Part 1	X										X
Timeline Followback			X	X	X	X	X	X	X	X	
Engagement in Substance-free activities	X										X
PEG for chronic pain	X		X	X	X	X	X	X	X	X	X
Brief Pain Inventory	X										X
EQ5D5L	X										X
PHQ-2 depression	X		X	X	X	X	X	X	X	X	X
GAD 2	X		X	X	X	X	X	X	X	X	X
Delay discounting	X										X
Opioid Purchase Task	X		X	X	X	X	X	X	X	X	X
Perceived social support	X										X
Buprenorphine-naloxone prescribed dose	X		X	X	X	X	X	X	X	X	X
Buprenorphine-naloxone dose log			X	X	X	X	X	X	X	X	X

This manuscript uses SPIRIT reporting guidelines [70]. A SPIRIT checklist is included as an additional file.

### Outcome measures

#### Primary outcome

The main outcome of the study is medication adherence, defined as attendance at the MOUD appointment with the buprenorphine care provider, and presence of buprenorphine in urine.

### Treatment moderators

Moderators that will be examined fall into three categories. Behavioral economics-related variables will be assessed at each study visit using self-report questionnaires. These include measures of delay discounting [45], opioid demand [47], engagement in substance-free activities [57], adherence self-efficacy [71], and locus of control [72].

Additionally, measures of mental health diagnoses will be examined via self-report measures, (Patient Health Questionnaire-2 [63] and Generalized Anxiety Disorder 2-item [60]) and transcription from the medical file. Further, we will measure opioid and other substance use using the Timeline Followback [73], measures of opioid cravings [67], and opioid use disorder criteria [44]. We will also assess adverse events (e.g., overdose) and pain [61].

### Study informatics

All data entry, processing, and management will occur via a relational database. All of the informatics structures within this system will operate in a client/server network environment and provide methods to secure data [33]. Only study personnel with appropriate access privileges will have access to the informatics system.

Data will be stored on a HIPAA-compliant cloud software. These datasets will be stored with read-only privileges to prevent any changes to the file. The statistician will be able to download the data as needed. Data will be coded and a codebook developed.

### Interventionist training and treatment fidelity

Interventionists will be master’s level counselors trained in the execution of the study interventions. Following didactic training on session content, interventionists will be required to “test out” of the intervention sessions by enacting them with the clinical supervisor of the study, who will serve as the practice patient.

All treatment sessions will be audio recorded in order to conduct treatment fidelity on 20% of all sessions (224 of a possible total 1120 intervention sessions recorded) by the clinical supervisor of the study.

### Participant timeline

Participants will be enrolled at the time of their first visit to the buprenorphine treatment provider. Participants will be seen up to four times in Stage 1 randomization at their next four provider visits. Assessments and intervention will occur at each of these visits. If a participant is re-randomized into Stage 2, they will be seen at the next four visits. Assessments and intervention will also occur at each of these visits. There will be a final follow-up assessment 8 months after enrollment.

### Study oversight

Investigative Team is comprised of the Principal Investigator and six Co-Investigators with specific expertise required for the execution of this trial. Two investigators provide medical oversight and safety review, three provide theoretical and cognitive behavioral oversight, and two provide data management and data acquisition oversight.

A data safety monitoring board (DSMB) unaffiliated with the study team and funding agency will evaluate procedures for participant safety and study futility. This board will report directly to the funding agency every 6 months throughout the funding period. The DSMB has no competing interests.

### Statistical analysis

#### Power analysis

When enrolling *N* = 280 patients (140 in each of the two arms in Stage 1), we are adequately powered for the following hypotheses/tests: For the intermediate outcome adherence after 4 physician visits (initial treatment), BSM and CM result both in about 50% adherence by end of Stage 1 (we have at least 80% power to reject our null hypothesis that the adherence rate is not exceeding 50% as long as the true adherence rate is not exceeding 63%; two one-sided tests for proportion in both initial treatment arms, family-wide type I error alpha controlled at 5% by a Bonferroni correction accounting for 10% attrition; power assessment utilized G*Power 3.1.9.2 [74]). Furthermore, for the primary outcome of buprenorphine-naloxone adherence by the end of Stage 2 and viewing the re-randomization of non-adherent patients in Stage 2 as integral part of the treatment regimen, we have 80% power to detect a difference in final (after Stage 2) adherence proportions of Cohen’s *h* = 0.72 (comparing initial treatments BSM vs. CM), an effect size between medium and large as outlined by Cohen [75] (two-sided test with type I error alpha = 0.05, accounting for 10% attrition).

The SMART design also allows us to address several questions relating to the embedded dynamic treatment regimens (DTRs). We expect to have sufficient numbers in even the smallest groups in Stage 2 to derive meaningful insights for future trials: We have at least 90% chance to have 26 individuals to enter the smaller of the re-randomized groups in Stage 2 in each of the initial arms, assuming a 50% non-adherence rate in the first-line treatment and accounting for 10% attrition (90% chance of at least 20 individuals with 40% non-adherence rate in first-line treatment; computations used specialized R-code provided by [76]). These secondary hypotheses include an assessment whether adding the respective other treatment (BSM and CM) is better than switching intervention for initially non-adherent patients. In addition, we will assess whether BSM followed by CM is different from CM followed by BSM for the non-adherent individuals after Stage 1. Note that such an assessment is based on a weighted-results approach due to the re-randomization in Stage 2 conditional on failure in Stage 1 [77, 78].

### General statistical procedures

The primary data analysis will follow an intent-to-treat analysis [79]. The principal approach with respect to missing data is foremost to make every effort to keep missing data to a minimum [80]. If missingness is unavoidable, contemporary multiple imputation [81, 82] will be used in the main analysis. We will initially summarize and compare participants’ anthropometric, demographic, and other baseline characteristics and will test for systematic differences in these for participants who do meet or do not meet adherence after Stage 1 and, again, Stage 2 using *t*-test/Wilcoxon-test, chi-square/Fisher’s exact test, and weighted approaches when indicated by the SMART design [77].

We will also conduct a descriptive heterogeneity of treatment effect analysis (HTE) [83] with respect to sex, gender, race, socioeconomic status, and age. We will test for HTE by modeling the interaction between treatment arm and covariate and declare HTE present if the interaction term is statistically significant at the 5% level based on a likelihood ratio test. We will conduct and report the HTE-analyses (including 95% confidence intervals) irrespective of the determination of a statistically significant overall effect to facilitate the planning of future clinical trials and subsequent meta-analytical approaches investigating possible sex-, gender-, or race-specific effects.

### Planned comparisons

Hypothesis 1: We predict that a higher percentage of those in the CM condition will be adherent after 4 weeks of intervention than those in BSM: Comparison of proportions and logistic regression with adherent yes/no as dependent variable includes baseline demographics, etc., as explanatory variables. We are adequately powered to test this hypothesis as well as the question whether there are differences in adherence after the 6-month maintenance period depending on initial treatment assignment.

Hypothesis 2: Based upon evidence of diminished effects of CM over time, we predict that those in CM (vs. BSM) will show decreases in adherence during the 8-month follow-up: Approach is as for Hypothesis 1 above but based on all entering the maintenance phase after being adherent for 4 weeks. We have adequate power only if the relative difference between these two groups is large (80% power to detect, e.g., 10% vs. 32% subsequent non-adherence, assuming stage-1 adherence proportions of *γ*_1_ = *γ*_2_ = 0.5 and accounting for 20% attrition; smaller differences can be detected if initial adherence proportions are higher or attrition is lower). Even here, we will report our findings including 95% confidence intervals to facilitate the planning of future clinical trials and subsequent meta-analytical approaches.

Hypothesis 3: We predict that the combined CM + BSM will have the highest treatment effect among those who are initially non-adherent, given the greater intensity of intervention. Comparison of the relevant final adherence proportions in the participants re-randomized, using Tukey’s Honest Significant Difference (HSD) procedure to adjust for the multiplicity in the pairwise comparisons. We expect to have a minimum of 23 individuals in the smallest of these re-randomized groups, but do not expect to be powered to authoritatively conclude about this hypothesis. We will report our findings including 95% confidence intervals to facilitate the planning of future clinical trials and subsequent meta-analytical approaches.

### Monitoring

The Principal Investigator (PI) will evaluate security of data and safety of participants monthly. Reports will be sent to the PI and statistician each month by the Study Coordinator regarding recruitment and retention.

### Data Monitoring Committee

A Data Safety Monitoring Board (DSMB) has been established for this study. While the interventions used in this pilot are believed to pose minimal risk, the high-risk nature of the study population (Opioid Use Disorder patients) makes additional protections a necessity. This committee comprised of four qualified persons independent of competing interests who will meet a minimum of once every 6 months. Types of credentials our DSMB members possess are as follows: (1) experience conducting behavioral intervention studies; (2) experience with treating opioid use disorder; and (3) biostatistical expertise in intervention studies. As DSMB members, the DSMB will review the reports sent by the study statistician to determine whether there is any corrective action, trigger of an ad hoc review, or stopping rule violation that should be communicated to the study investigator, the IRB, or the funder. The DSMB will be headed by a Chair member who will send the DSMB’s recommendation to approve, halt, or discontinue the study to the funder.

### Interim analyses

Interim analyses will be conducted solely for the DSMB reports and annual reports to the funder. The analyses include subject accrual, adverse event (AE), and serious adverse event (SAE) rates by arm, overall retention and retention by arm, adherence by study arm, and stopping rules regarding statistical power implications of drop-outs and missing data. All of these analyses with the exception of adherence by study arm will be available to all investigators and DSMB members. The investigative team, except for the statistician compiling the report, will not have access to adherence by arm since it is a primary outcome.

### Harms

A physician Co-Investigator will be responsible for overseeing AE and SAE reporting. Research coordinators will take initial reports from participants and the physician will review for safety and accuracy and recommend follow-up as needed. All AEs and SAEs will be reviewed within five business days. Any SAEs possibly related to study intervention will be reported to the IRB, DSMB, and funding agency within five business days.

### Auditing

While the funder does not plan regular audits, the study team is prepared for any possible audits from the funder or the approving IRB.

### Ethics and dissemination

#### Research ethics approval

This study was approved by the Institutional Review Board at the University of Tennessee Health Science Center (FWA00002301). Informed consent will be obtained from all participants before research procedures commence.

### Protocol amendments

Protocol amendments of any nature will be submitted to the IRB for approval. Major protocol changes will also be approved by the funder before implementation.

### Consent

Informed consent will be obtained from all participants before any research procedures take place. The consent form is approved by the reviewing IRB and contains all basic and additional elements of informed consent as regulated by 45CFR46.

Trained study staff will obtain informed consent primarily in-person at the participants’ initial buprenorphine provider visit. However, the IRB approved an alteration of consent for electronic consent when needed. The informed consent discussion takes place via telephone while the participants views and signs the consent electronically.

### Confidentiality

All personally identifying information (PII) and personal health information (PHI) needed for recruitment, study involvement, and tracking will be obtained and maintained by the project personnel in a secured server with limited access. All computer files and systems are password protected and accessible by authorized personnel only. Data entry and transfer will be performed by the study staff shared only with those persons authorized to have access.

All paper records will be locked in a secure file cabinet behind at least two locked doors. Only necessary staff will have access.

The datasets from the study will not contain any PHI. Participants will have an assigned study ID number and their data will be associated with this number only.

### Data access

The final trial dataset will be available initially to the study’s Investigators. After the main outcomes are published, public access will be provided.

This project was funded through the NIH HEAL (Helping End Addiction Long-term) Initiative, which requires award recipients to share data via the HEAL Initiative Data Ecosystem in compliance with the HEAL Initiative Public Access and Data Sharing Policy, which is in line with the new NIH policy for Data Management and Sharing. Data shared here will be de-identified.

### Dissemination policy

#### Trial results

The results of this study will be disseminated by publication of peer-reviewed manuscripts. This project will comply with NIH public access policy, and we plan to make our peer-reviewed manuscripts available to other researchers and to the public at the NIH National Library of Medicine’s PubMed Central immediately after the final date of journal publication.

### Authorship

Other researchers who are interested in using this data for publication will be able to submit a request to the investigative team.

### Reproducible research

The protocol for this study will be published on ClinicalTrials.gov at the completion of the study. The dataset and statistical code will be available by request to the investigative team.

Trial*** status.***

Recruitment for this trial began on February 1, 2022. We anticipate enrollment to be completed by the end of 2023. At the time this manuscript is being written, protocol version 2.0 from 11/15/2022 is in use.

## Discussion

The innovative design of this clinical trial that will compare two adherence-promoting interventions to increase adherence to buprenorphine-naloxone medication is likely to have a significant impact on engagement of MOUD patients in treatment and may inform practices about the use of adjunctive intervention for medication adherence during treatment initiation, a known period of high risk for those with OUD.

Further, the use of a SMART design will test multiple levels of intervention for individuals who may not have initial MOUD adherence success. For those who are not adherent in Stage 1, sequential intervention resources will be provided to examine if a different intervention or a more intensive (combined) intervention is needed to assist these individuals in their treatment journey.

Through the extension of successful intervention into a maintenance phase, we will be able to examine long-term effects across initiation and maintenance phases of MOUD. This allows us to examine whether adapting adjunctive intervention for those who do not initially adhere is a worthwhile strategy. Results from this work will also directly inform the equipoise surrounding the long-term use of CM for medication adherence, a strategy that has demonstrated short-term gains in promoting opioid abstinence but diminished effects thereafter.

Finally, this study will be able to examine how delay discounting, a malleable characteristic known to change over the phases of active substance use disorder and recovery, changes with these different forms of intervention. While CM directly supplements a need for immediate reward, BSM works explicitly to address delaying immediate rewards in favor of larger gains that come with MOUD adherence and fulfilling named goals. It is possible that the action of these intervention methods will be apparent through examination of delay discounting throughout the trial.

This trial is designed to examine the comparative and sequential effects of two forms of adjunctive intervention to promote MOUD adherence. It is possible that through this work, characteristics of risk for opioid use will add to the body of evidence that allows practitioners to make the most out of limited resources and reach as many patients as possible to encourage treatment adherence.

## Supplementary Information

**Additional file 1.** Appendix: Model Consent Form.

## Data Availability

Available upon request.
