# Supplementary material for: Use of a sequential multiple assignment randomized trial to test contingency management and an integrated behavioral economic and mindfulness intervention for buprenorphine-naloxone medication adherence for opioid use disorder
Source: Trials. 2023 Mar 29;24:237. doi: 10.1186/s13063-023-07102-9 (PMC10061866; doi:10.1186/s13063-023-07102-9)
Supplement: Supplementary file 1 — Additional file 1. Appendix: Model Consent Form. [file 13063_2023_7102_MOESM1_ESM.docx]

**Appendix: Model Consent Form**

**1. KEY INFORMATION:**

You are being given the opportunity to participate in this research study. The purpose of this consent form is to help you decide if you want to be in the research study.

The purpose of this study is to compare the effectiveness of two active treatments for medication-assisted treatment (MAT) adherence. All research study subjects will be receiving standard medication to address Opioid Use Disorder (OUD) through the clinic. The two treatments, Contingency Management (CM) and Brief Motivational Interviewing + Substance-Free Activities Session + Mindfulness (BSM), are additional treatments that may help you adhere to your medication received from your doctor and decrease the likelihood that you will return to opioid use.

If you enroll, you will be randomly assigned to one of these two groups. CM is a form of treatment where incentives are provided for certain behaviors. In this study, CM participants will receive gift cards for drug screens positive for buprenorphine. BSM participants will have one-on-one sessions with a counselor to discuss their goals, how to reach these goals, and the importance of drug-free activities.^31,32^

**Procedures:**

In this study, we will randomize subjects between different treatments to compare their success rate and safety. We will also be collecting data from your medical record as you complete visits for your clinical care and asking you to complete some additional questionnaires about your opioid and other drug use.

You will be randomly assigned (like the flip of a coin) to receive Contingency Management (CM) or Brief Motivational Interviewing + Substance-Free Activities Session + Mindfulness (BSM). Everyone will receive standard medical treatment from the clinic, but the research component of the study involves two treatment interventions that are not yet proven to be effective in helping patients to avoid opioid use. The investigator will not be the person who decides which you receive. A computer program that gives random numbers will be used to decide which you receive. It is not known which treatment, if either, is better.

You may be randomly assigned to a new treatment group during the first 2 months of the study. This will occur based on your progress in treatment. For full description of reasons you may be reassigned, see the DETAILED PROCEDURES TO BE FOLLOWED section below.

If you are reassigned, you will either be switched to the other treatment or a combination of the two treatments. For example, if you were originally randomized to BSM and you are reassigned, you will be randomized to either CM+BSM or CM. If you were originally randomized to CM and you are reassigned, you will be randomized to either CM+BSM or BSM. It is not known if the combination treatment is better, worse, or the same as the other two treatments used alone. If you are reassigned, the investigator will not be the person who decides which you receive. A computer program that gives random numbers will be used to decide which you receive.

If you decide to receive care at a different clinic but still wish to participate in this study, we will ask that you either 1) sign a release so we can receive your toxicology screen results or 2) will collect saliva samples from you for toxicology results. This is so that we can continue to monitor your adherence and give you the incentives for taking your buprenorphine medication.

Your participation in this study will last 8 months. If you like your treatment and wish to continue, we can see you monthly through the first 8 months of your treatment. This additional treatment will not involve recording of any research data. All visits will take place in person when you come in for your regular physician visits.

**The following procedures are being** **performed for research purposes only**:

- Copying information such as your medical history, urine screen results, and current diagnoses from your medical record;
- Between 6–10 visits with study staff for treatment and/or assessment;
- 21 questionnaires;
- Saliva drug screening (only if you change treatment clinics).

For a detailed explanation of the procedures, refer to the section of this consent form entitled, DETAILED PROCEDURES TO BE FOLLOWED.

**Risks:**

There are no physical risks associated with this study; however, answering some of the questionnaires and topics discussed in the sessions may cause feelings of discomfort of cause troublesome feelings or emotions.

**Benefits:**

Your adherence to medication-assisted treatment may improve while you are in this study; however, this cannot be promised.

The results of this study may help people with Opioid Use Disorder in the future to be able to better adhere to treatment.

**Alternatives:**

You may receive additional treatment for Opioid Use Disorder without participating in this study.

If you decide not to enter this study, there are other choices available such as one-on-one counseling or group therapy. Ask the study doctor to discuss these alternatives with you. You do not need to be in this study to receive treatment for your condition.

You will receive medical treatment for Opioid Use Disorder whether or not you participate in the study.

**Voluntary Participation:**

Your participation in this research study is voluntary. You may decide not to participate or you may leave the study at any time. Your decision will not result in any penalty or loss of benefits to which you are entitled.

If you are a student of UTHSC, participating or not participating in this study will in no way influence your grade in any course. If you are a resident or fellow of UTHSC, participating or not participating in this study will in no way influence your academic standing. If you are an employee of UTHSC or University Clinical Health, participating or not participating in this study will not affect your employment status.

**2. DETAILED PROCEDURES TO BE FOLLOWED:**

340 subjects will be participating in this study. The study will take place at

- University Clinical Health Center for Addiction Science located at 6401 Poplar Ave, Suite 500, Memphis TN 38119, and
- Christ Community Health Services located at 3362 S. 3^rd^ St, Memphis, TN 38109, and
- Alliance Healthcare located at 1200 Peabody Ave, Memphis, TN 38104, and
- Integrated Addiction Care Associates (IAC/Vertava Health), 6300 Summer Knoll Cir, Suite 101, Bartlett, TN, 38134.
- UTHSC Department of Preventive Medicine, 66 N. Pauline St, 5^th^ Floor, Memphis, TN 38163.

Visit 0: Baseline (this will take an additional 40 min. at your doctor visit):

- Completing the baseline questionnaire including age, gender, race, and opioid use history. A total of 20 questionnaires will be administered.
- Information about your opioid prescription, drug screen results, mental health disorder diagnoses, and other substance use disorder diagnoses will be copied from your medical record.

Visits 1-4: (this will take an additional 10-30 min. at your first 4 return doctor visits, starting one week from today):

- Randomization #1 into assigned treatment group by a computer program (Visit 1 Only)
  - The CM group will receive incentives in visits 1-4 if your urine screen shows you are taking your buprenorphine medication.
  - The BSM group will participate in a discussion of short and long-term goals, how to reach these goals, and the importance of engaging in enjoyable and goal-directed drug-free activities.
- Completing 11 questionnaires including opioid use, opioid craving and withdrawal symptoms, and medication logging
- Information about your opioid prescription, drug screen results, mental health disorder diagnoses, and other substance use disorder diagnoses will be copied from your medical record
- CM or BSM treatment
- Participants in the CM arm will get to spin a wheel to win either a $50, $75, or $100 gift card at each visit if the following conditions are met: 1) attend physician visit and 2) toxicology screen results are positive for buprenorphine
- Participants in the BSM arm will engage in a counseling-style treatment focused on developing personal goals, engagement in substance-free activities, and mindfulness activities.
- Staff will assess for adherence using your drug screen results and visit attendance. If any of the following situations occur during the first 4 visits, we will assume your group assignment is not an optimal fit for you, and you will be re-randomized into a different treatment or additional treatment. If you are re-randomized, you will repeat your opportunity to complete visits 1–4.
  - Two or more follow-ups with negative for buprenorphine (not adhering to medication-assisted therapy)
  - Two or more missed follow-ups

Visits 5-8: Re-randomized participants only (this will take an additional 10-30 min. at your first 4 return doctor visits

- If you are re-randomized, you will repeat your opportunity to complete visits 1-4.
- Completing 11 questionnaires including opioid use, opioid craving and withdrawal symptoms, and medication logging
- Information about your opioid prescription, drug screen results, mental health disorder diagnoses, and other substance use disorder diagnoses will be copied from your medical record
- CM, BSM, or CM+BSM treatment (described above)

Visit 5: 8-month follow-up (this will take an additional 30 min. at your doctor visit

- Completing 17 questionnaires including opioid use, opioid craving, and withdrawal symptoms.
- Information about your opioid prescription, drug screen results, mental health disorder diagnoses, and other substance use disorder diagnoses will be copied from your medical record.

If you decide to receive care at a different clinic but still wish to participate in this study, we will ask that you either 1) sign a release so we can receive your toxicology screen results or 2) will collect saliva samples from you at each for toxicology results. This is so that we can continue to monitor your adherence and give you the incentives for taking your buprenorphine medication. The saliva will be tested immediately and then discarded.

Your participation in this research study may be stopped by the study doctor without your consent for any of the following reasons:

- If you do not show up for visits
- If you do not follow the study doctor’s instructions
- If you decide to stop taking part in this research study, you should tell your study doctor, and any information that you have already provided will be kept in a confidential manner.

You may ask that your identifiable samples be destroyed.

**3. RISKS ASSOCIATED WITH PARTICIPATION**:

There is a risk that your private identifiable information may be seen by people not involved in the research (such as if a researcher’s computer is stolen or an electronic database is hacked). However, we will use very careful security measures (such as locks on file cabinets, computer passwords, etc.) to minimize the chance that any unauthorized persons might see your confidential information.

The research may involve risks to you which are currently unforeseeable. You will be told about any new information that might change your decision to be in this study. You may be asked to sign a new consent form if this occurs.

**Intervention/Treatment Sessions:** Completion of the BSM sessions may make you feel uncomfortable or cause troublesome feelings or emotions. You may take a break at any time during the study.

**Questionnaires/Surveys:** Completion of the questionnaires may make you feel uncomfortable or cause troublesome feelings or emotions. You may refuse to answer any of the questions and you may take a break at any time during the study.

**Audio Recording:** Having your voice recorded may make you feel uncomfortable. You may take a break during any time of the study. There is also a potential risk of loss of confidentiality that someone who listens to your audio recording might identify you. If you opt-out of having your session recorded, you may continue to participate in the session.

**4. CONFIDENTIALITY:**

**Research records/specimens**

All your paper research records will be stored in locked file cabinets and will be accessible only to research personnel and those entities named below in this section, except as required by law (such as reports of child abuse, plans to commit suicide, etc.).

All your electronic research records will be kept on an encrypted computer where your information is replaced with a code and password only known to the research personnel, except as required by law (such as reports of child abuse, plans to commit suicide, etc.).

If you choose to participate in the saliva sample due to changing clinics, your sample will be labeled with a code during testing then will be immediately destroyed.

A master key/list which links your name with the code on your research record will be maintained at UTHSC.

Your private information collected as part of this research, even if identifiers are removed, will not be used or distributed for future research studies.

**Medical Records**

Information about your participation in this study or the results of procedures performed in this study will be placed in your medical record; as such, this information could be made available to your employer or insurer.

**Presentations/Publications**

While individual details about your case might be provided in publications or presentations about this research, they will not be discussed in a way that would allow you to be individually identified as a participant.

**Limits to Confidentiality**

Information obtained during the course of the study, which in the opinion of the investigator(s) suggests that you may be at significant risk of harm to yourself or others, may be reported to a third party to protect the rights and welfare of those at potential risk.

**Authorization to Use and Disclose Protected Health Information for Research Purposes** Under federal privacy regulations, you have the right to decide who can review and copy your identifiable health information (called “protected health information” or PHI). PHI collected in this study may include information such as:

- Past and present medical records
- Records about your study visits
- Records about phone calls made as part of this research
- Research records

By signing this consent form, you are giving your permission for the study doctor and the study staff to get your PHI from your doctor and/or facilities where you have received health care. They may also share your PHI with:

- The Institutional Review Board (IRB) at the University of Tennessee Health Science Center
- University Clinical Health
- Christ Community Health Services
- Alliance Healthcare
- Integrated Addiction Care

Your PHI will only be used and/or given to others:

- To do the research
- To study the results
- To see if the research was done correctly

Your PHI will be used until the study is completed.

You may withdraw or take away your permission to use and disclose your PHI at any time. You do this by sending written notice to the study doctor. If you withdraw your permission, you may not be able to stay in the study.

When you withdraw your permission, no new PHI will be gathered after that date. However, information that has already been gathered may still be used and given to others. The federal regulations allow you to review or copy your PHI that is used in this study.

**Certificate of Confidentiality**

This research is covered by a Certificate of Confidentiality from the National Institutes of Health. The researchers with this Certificate may not disclose or use information or documents that may identify you in any federal, state, or local civil, criminal, administrative, legislative, or other action, suit, or proceeding, or be used as evidence, for example, if there is a court subpoena, unless you have consented for this use. Information and documents protected by this Certificate cannot be disclosed to anyone else who is not connected with the research except when: (1) there is a federal, state, or local law that requires disclosure (such as to report child abuse or communicable diseases); (2) you have consented to the disclosure, including for your medical treatment; or (3) the materials are used for other scientific research, as allowed by federal regulations protecting research subjects.

The Certificate of Confidentiality will not be used to prevent disclosure as required by federal, state, or local law of child abuse or neglect, or harm to self or others.

You should understand that a Certificate of Confidentiality does not prevent you from voluntarily releasing information about yourself or your involvement in this research. If you want your research information released to an insurer, medical care provider, or any other person not connected with the research, you must provide consent to allow the researchers to release it.

**5. COMPENSATION AND TREATMENT FOR INJURY:**

You are not waiving any legal rights or releasing the University of Tennessee, University Clinical Health, Christ Community Health Services, Alliance Healthcare, Integrated Addiction Care, or the agents of either, from liability for negligence. In the event of physical injury resulting from research procedures, the University of Tennessee and University Clinical Health do not have funds budgeted for compensation for medical treatment. Therefore, the University of Tennessee and University Clinical Health do not provide for treatment or reimbursement for such injuries.

If you are injured or get sick as a result of being in this study, call the study doctor immediately. The study doctor will provide acute medical treatment, and will provide you with a subsequent referral to appropriate health care facilities.

If you are injured or get sick as a result of being in this study, you and/or your insurance will be billed for the costs associated with this medical treatment.

No compensation will be available to you for any extra expenses that you may have as the result of research-related physical injuries, such as additional hospital bills, lost wages, travel expenses, etc*.*

No compensation will be available to you for any non-physical injuries that you may have as a result of research participation, such as legal problems, problems with your finances or job, or damage to your reputation.

**6. QUESTIONS:**

Contact the Research Coordinator, at xxx-xxx-xxxx if you have questions about your participation in this study, or if you have questions, concerns, or complaints about the research.

If you feel you have had a research-related injury, contact (clinic) at xxx-xxx-xxxx and you will be directed to an answering service during non-business hours.

You may contact UTHSC IRB Director, at xxx-xxx-xxxx, or visit the IRB website if you have any questions about your rights as a research subject, or if you have questions, concerns, or complaints about the research.

**7. PAYMENT FOR PARTICIPATION:**

All payment is in the form of your choice of a Kroger or Amazon gift card. Payment for your participation in assessments and visits will be different depending on which group you have been assigned to. An explanation for each group is below:

BSM Group: participants will receive a $50 gift card upon completion of each of the 4 assessment visits as well as a $50 gift card upon completion of the baseline (today’s) visit. Participants will also receive a $100 gift card upon completion of the 8-month follow-up. Participants in this arm may receive up to $350.

CM Group: participants will receive a $50 gift card upon completion of each of the 4 assessment visits. At these visits, participants who attended their physician visit and whose drug screens are buprenorphine-positive will spin a wheel and receive an additional $50, $75, or $100 gift card. Participants will receive a $100 gift card upon completion of the 8-month follow-up. Participants in this group may receive up to $750.

BSM+CM Group: participants who are re-randomized to this group will receive a $25 gift card upon completion of each of the 4 assessment visits. At these visits, participants who attended their physician visit and whose drug screens are buprenorphine-positive will spin a wheel and receive an additional $50, $75, or $100 gift card. Participants will receive a $100 gift card upon completion of the 8-month follow-up. Participants in this group may receive up to $750.

You will receive a gift card at the end of each of your visits. If you complete any of your visits over the phone due to COVID or other circumstances, we will mail you the gift card.

**8. COSTS OF PARTICIPATION:**

There are no costs to you for participating in this study. Tests and procedures that are done only for research purposes will not be billed to you or your insurance company.

**9. FUTURE CONTACT:**

If we lose contact with you during the study for any reason (your phone number changes; your physical or email address changes; you are not responding to our attempts to contact you about your continued participation; etc.), we will attempt to find you or make contact with you in the following ways:

- The phone number(s) you provided to us will be called, but if you are not the person who answers, we will not say the title of the study or the fact that you are/were participating in a study.
- A text message will be sent to the phone number(s) you provided to us requesting that you call us.
- A letter will be sent to the address(es) you provided to us, but neither the return address nor any markings on the envelope will identify the title of the study or the fact that you are/were participating in a study.
- An email will be sent to the address you provided to us.
- The alternate contact person you provided to us will be contacted. We will only tell the person we’re calling about a research study if you give us permission to do so.

Put your initials on one of the lines below:

- _______We CAN attempt to find/contact you in the above ways.
- _______We MAY NOT attempt to find/contact you in the above ways.

Sometimes we wish to keep your contact information, medical diagnosis, and other health information in order to contact you in the future and tell you about other studies in which you might be eligible to participate.

Put your initials on one of the lines below:

_______We CAN keep your contact information and health information to ask you about participating in future studies.

_______We MAY NOT keep your contact information and health information to ask you about participating in future studies.

**10. CONSENT OF SUBJECT:**

You have read or have had read to you a description of the research study as outlined above. The investigator or his/her representative has explained the study to you and has answered all the questions you have at this time. You knowingly and freely choose to participate in the study. A copy of this consent form will be given to you for your records.

___________________________________________ ___________ _________

**Signature of Research Subject (18 years +)** **Date Time**

___________________________________________

**Printed Name of Adult Research Subject**

____________________________________________ ___________ __________

**Signature of Person Obtaining Consent** **Date Time**

____________________________________________

**Printed Name of Person Obtaining Consent**

In my judgment, the subject has voluntarily and knowingly given informed consent and possesses the legal capacity to give informed consent to participate in this research study.

__________________________________________ _______ _______

**Signature of Investigator** **Date Time**
